# A System of Emotion Recognition and Judgment and Its Application in Adaptive Interactive Game

**DOI:** 10.3390/s23063250

**Published:** 2023-03-19

**Authors:** Wenqian Lin, Chao Li, Yunjian Zhang

**Affiliations:** 1 School of Media and Design, Hangzhou Dianzi University, Hangzhou 310018, China; 2 College of Computer Science and Technology, Zhejiang University, Hangzhou 310027, China; 3 College of Control Science and Technology, Zhejiang University, Hangzhou 310027, China

**Keywords:** emotion judgment system, adaptive interactive game, set of optimal signal features, sensor

## Abstract

A system of emotion recognition and judgment (SERJ) based on a set of optimal signal features is established, and an emotion adaptive interactive game (EAIG) is designed. The change in a player’s emotion can be detected with the SERJ during the process of playing the game. A total of 10 subjects were selected to test the EAIG and SERJ. The results show that the SERJ and designed EAIG are effective. The game adapted itself by judging the corresponding special events triggered by a player’s emotion and, as a result, enhanced the player’s game experience. It was found that, in the process of playing the game, a player’s perception of the change in emotion was different, and the test experience of a player had an effect on the test results. A SERJ that is based on a set of optimal signal features is better than a SERJ that is based on the conventional machine learning-based method.

## 1. Introduction

Emotion plays an important role in daily life and is a critical factor that affects the process of an individual’s cognitive, communication, and decision-making abilities. Physiological signals, such as skin electricity, electrocardiogram, pulse wave, and facial electromyogram, can be used to recognize and judge individuals’ emotions [1]. In the past few years, research has reported on the recognition and judgment of emotions based on physiological signals. The fusion of multiple emotional modalities was proposed by Khezri et al. [2] to improve the performance of an emotion judgment system. In the presented emotion recognition system, recorded signals with the formation of several classification units identified the emotions independently, and considerable improvement was obtained. A new approach for the empirical identification of affected regions, which was based on skin conductance, was put forward by Liapis et al. [3]. Their findings identified the regions in the valence–arousal rating space that might reliably indicate self-reported stress while using interactive applications. A new method recognizing the emotional state of six individuals was given by Yoo et al. [4]; the method possessed good performance accuracy and could make a distinction between one emotion and other possible emotional states. Yang et al. [5] put forward a new method, which was based on skin conductance, that classified the emotion image based on the electroencephalography signals; the new method bridged the emotion gap by building a relationship between experiential information and the expected emotion experience of the viewer, and the results showed that the method could bring about a pleasant experience. Jang et al. [6] experimentally evaluated the dependability of physiological signal changes initiated by multiple emotions by measuring six basic emotions; they indicated that the physiological signals based on heart rate, skin conductance, and blood volume pulse were more reliable than those evaluated at baseline. An algorithm was put forward by Sepulveda et al. [7] to ameliorate the emotion recognition extracted from electrocardiogram signals using wavelet transform for signal analysis, and the algorithm, when combined with wearable devices, proved to be effective for classifying emotion. In order to recognize emotion based on multimodal physiological signals, Zhang et al. [8] proposed a deep-fusion framework, which displayed higher class separability in emotion recognition, and this framework was more effective in subject-independent emotion recognition than other fusion methods. A framework of multimodal emotion classification using multichannel physiological signals was proposed by Yan et al. [9]; they pointed out that it was significant to develop adaptive decision fusion strategies in the process of emotion classification.

Although some achievements have been made in recognizing and judging emotions from physiological signals, based on the above research, there is still room for improvement in the accuracy of judgment and the universality of application, for example, in the field of interactive games.

There is an increasing interest in creating games that are based on interaction technology. Although interactive games belong to the category of human–computer interaction, they are different from general human–computer interaction in the following aspects: (1) Compared with general human–computer interaction, interactive games pay more attention to the process of interaction rather than the result of interaction; (2) the meaning and purpose of general human–computer interaction are determined by the user’s purpose and task, while those in interactive games are determined by the purpose and operation form of the game itself; (3) general human–computer interaction is more stable and focuses on the durability of functions to consolidate the user experience, while interactive games focus on waking up a series of user experiences; and (4) whether in interactive content or control systems, interactive games have ample room for innovation.

For a long time, game developers have tried to apply physiological signals to the process of playing games, hoping that a player’s experiential state could be captured in real time when a player realized the capture, so as to enhance the interest and intelligence of the game. As early as 1984, CalmPute designed a device called CalmPrix that operated racing games based on the skin electrical signals. In 1998, Nintendo released the physiological sensor Teris64. In 2010 and 2011, Nintendo released Wii accessories based on physiological signal sensors. In 2011, Ubisoft also announced the development of similar products. However, these products have not been widely used. One of the reasons is that the equipment is too cumbersome and complex to wear, and another reason is that it does not conform to the operation habits of players in some aspects.

Lv et al. [10] designed and evaluated a touchless motion interaction technology and developed three primitive augmented-reality games with 11 dynamic gestures. Players interacted with the augmented-reality games using hand/feet gestures in front of the camera, which triggered the interaction event to interact with the virtual object in the scene. Vachiratamporn et al. [11] analyzed the affective states of players prior to and after witnessing a scary event in a survival horror game by collecting the player-affect data through their own affect annotation tool that allowed the player to report his affect labels while watching his recorded game play and facial expressions; the results showed that players were likely to get more fearful of a scary event when they were in the suspense state, and heart rate was a good candidate for the detection of player affect. Du et al. [12] presented an approach to detect a subject’s emotions using heartbeat signals and facial expressions, and the approach had high accuracy and less computational time for four emotions when playing different games. Baldassarri et al. [13] put forward two kinds of interactive games to promote communication and attention experience; one is to consider emotions to measure a user’s attention, concentration, and satisfaction, and the other is to use a tangible desktop to promote cognitive planning. Kalantarian et al. [14] proposed a method of automatically extracting emotion marker frames from a game and training a new emotion classifier to get over the limited function of the existing emotion recognition platform for children with autism spectrum disorders. Sekhavat et al. [15] studied the degrees to which the expression of the manifested emotions of an opponent could affect the emotions and, consequently, the game-play behavior by performing a comprehensive user study to estimate the emotions of players in emotion-aware versus emotion-agnostic game versions.

Nacke et al. [16] divided physiological signals into directly controllable and non-directly controllable signals and then asked the subjects to rate the game experience with and without controlled signals, respectively. The results showed that the effect of the game experience with directly controllable signals was better than that without controlled signals. However, there is a lack of research on the effect of the game experience with non-directly controllable signals. In addition, in terms of interactive game design, designers often deliberately encourage players to consciously change their emotional state through equipment and testing, which affects the player’s experience. In the design of this paper, the relationship between the game and the player is exchanged. Instead of letting players deliberately adapt to the game, the game is designed to automatically change the level according to the player’s emotional state and to adapt to the player’s emotional changes so as to increase the self-adaptability and the fun of the game and enhance the intelligence and naturalness of the interaction when players are not aware of it. Arendse et al. [17] evaluated the framework using player action data from the platforming game Super Mario Bros, and the results that were based on the presented framework were better than the existing work. Svoren et al. [18] built a dataset that consisted of demographic data assembled from ten participants playing Super Mario Bros and showed that the video game, together with facial expressions, could be used to predict the blood volume pulse of the subject. Granato et al. [19] predicted the subjects’ emotions during video game sessions and indicated that the obtained results could improve the game design. Izountar et al. [20] proposed a VR-PEER adaptive exergame system and developed a virtual reality-based serious game as a case study. The test results showed that fifteen participants expressed the usefulness of the system in motor rehabilitation processes. Kandemir and Kose [21] presented improved human–computer interaction games in attention, emotion, and sensory–motor coordination, and specially designed the interface and the difficulty levels of the games for the use of people from different age groups and with different disabilities. The tested results showed that the games had a positive effect on children. Penev et al. [22] examined the engagement level and therapeutic feasibility of a mobile game platform for children with autism by designing a mobile application, GuessWhat, which delivered game-based therapy to children in home settings through a smart phone; the tested results showed that the GuessWhat mobile game was a viable approach for the efficacious treatment of autism and further support for the possibility that the game could be used in natural settings to increase access to treatment when barriers to care exist.

Therefore, this paper consists of the following three parts. (1) A system of emotion recognition and judgment is built by collecting the change in physiological signals induced by emotional change and obtaining a set of optimal signal features. (2) The test on the above system is performed by 10 subjects playing the game Super Mario. The player’s emotional trend is triggered by the special events in the game. Meanwhile, the non-directly controllable physiological signals are detected to assess the effect of the game experience. (3) To illustrate the advantages of the optimal signal features, the emotional trend changes produced by the emotion recognition and judgment system based on the set of optimal signal features and based on the conventional machine learning-based methods are compared.

## 2. Emotion Recognition

**Figure 1:**
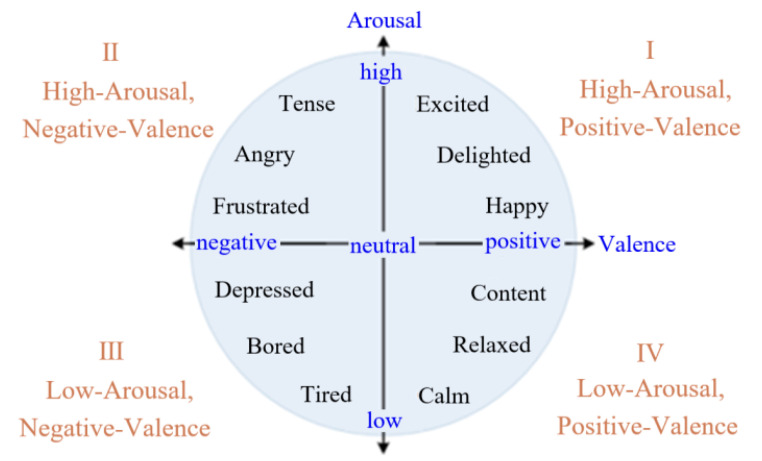
Valence–arousal model.

Emotions should be evaluated and classified before recognizing and judging them. The widely used valence–arousal (V-A) model is usually used to evaluate and classify emotions. In the V-A model, V and A represent the degree of emotional pleasure and emotion arousal, respectively [23]. Based on the discrete emotion classification, the emotions of fatigue, tension, happiness, and depression are first designated using the extracted four poles of emotion classification, which then is extended from four poles into a plane where, as shown in Figure 1, the four quadrants express, respectively, the high-arousal and positive-valence (quadrantⅠ: HAPV), high-arousal and negative-valence (Ⅱ: HANV), low-arousal and negative-valence (Ⅲ: LANV), and low-arousal and positive-valence (Ⅳ: LAPV).

## 3. Emotion Judgment

The establishment of an emotion recognition and judgment system through physiological signals includes the following basic steps.

### 3.1. Signals of Skin Electricity and Pulse Wave

In a system of emotion recognition and judgment, individuals’ physiological signals usually consist of pulse wave, skin electricity, electrocardiogram, and facial electromyogram. Which physiological signals should be used depends on the specific situation. Skin electrical signal and pulse wave signal are used in the present study. Since the skin electrical signal is easily interfered with by other signals inside the human body in processing, the noise interference generated by the hardware itself should be removed with the following formula:

(1) $$\begin{array}{l} f(t)=\mathrm{Serial}\_P\_R-\\ \frac{[(2014+2\times \mathrm{Serial}\_P\_R)\times 10,000]}{\left(512-\mathrm{Serial}\_P\_R\right)}, \end{array}$$

in which “Serial_P_R” is the data of the skin electrical signal, and the numbers are the debugged data based on the hardware properties.

In order to improve the performance of the computer analysis and processing, the discrete wavelet transform is used to denoise the physiological signals and decompose the signals into different frequency bands with low-pass and high-pass filtering.

The skin electrical signal is decomposed in three layers and then denoised using the wdencmp function in MATLAB; finally, all segments of the signal are normalized within the value range from 0 to 100.

The key factors to reflect the pulse wave signal are main wave, dicrotic anterior wave, dicrotic notch, and dicrotic wave. The amplitudes involved are main, dicrotic anterior, dicrotic notch, and dicrotic wave. The time refers to the time from the starting point of the waveform period to the lowest point of the dicrotic notch and to the peak c point of the main wave and the period of one waveform.

The pulse wave signal is smoothed and filtered using the Butterworth low-pass filter, which has the characteristics that the frequency response curve in the pass band is flat to the maximum without ripple and gradually drops to zero in the stop band:

(2) $$H(u,v)=\frac{1}{1+{\left[D(u,v)/D_{0}\right]}^{2n}}$$

The low-pass cut-off frequency is set to 10 Hz. The relevant parameters of the pulse wave signal are normalized after filtering.

### 3.2. Dimensionality Reduction in the Original Signal Feature

Dimensionality reduction in the original physiological signals is implemented with principal component analysis (PCA) because the direct fusion of the original physiological signals results in too much computation. Dimensionality reduction leads to a high efficiency and precision in the classification of emotion recognition. In the process of PCA, the weight of the original feature of the physiological signals is first calculated in the principal component, and then the weight threshold of each feature is taken as the criterion for choosing the feature. The original features with a larger weight than the threshold are chosen to form a new subset of the optimal features.

The method of Pearson correlation coefficient (PCC) is employed to determine the relation of the emotional interval, and these features are based on the subset of the optimal features. The PCC is performed on the features of four kinds of emotion trends and employed to extract the significance P of the features; then, the threshold of the optimal features correlated with four kinds of emotional trends is obtained according to the correlation coefficient and significance P of the features. In the present study, the optimal features are composed of “BpNN50”, i.e., the percentage of the main pulse wave interval larger than 50 ms, “range” of the skin electrical signal, and “1dmean”, i.e., mean value of the first order difference of the skin electrical signal. “BpNN50”, “range”, and “1dmean” are defined as follows:

(3) $$\mathrm{BpNN}50=\frac{\mathrm{count}\left|x_{i+1}-x_{i}\right|>50\mathrm{ms}}{N-1},i=1,\cdots ,N-1$$

(4) $$\begin{array}{c} \;\mathrm{range}=\max(x)-\min(x)\\ 1\mathrm{dmenn}=\frac{1}{N-1}\sum_{i=1}^{N-1}(x_{i+1}-x_{i}) \end{array}$$

where *x* is the discrete signal value, *I* is the *i*th signal, and *N* is the total number of signals.

### 3.3. Model of Emotion Judgment

**Figure 2:**
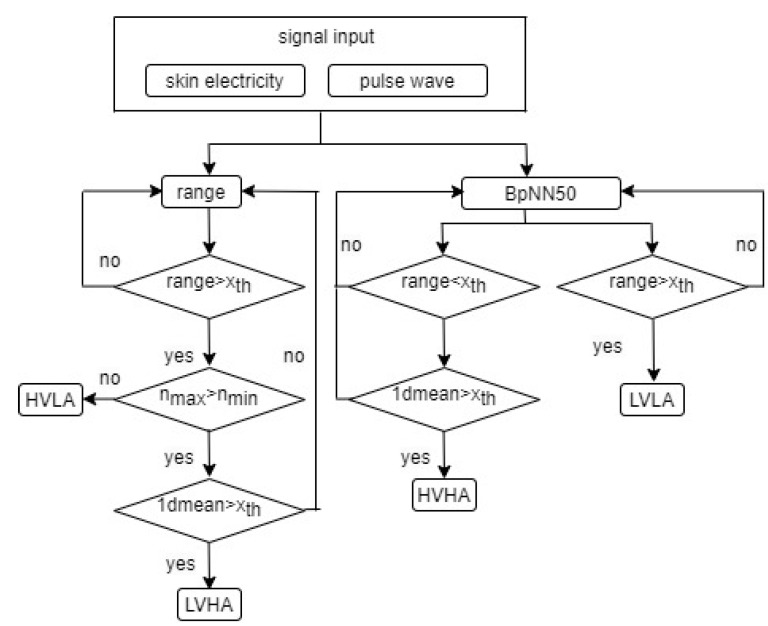
Model of emotion judgment.

According to the above description, the skin electrical signal and pulse wave signal are selected as the physiological signals to establish the emotion judgment system. The specific operation process is shown in Figure 2, where “BpNN50” is the percentage of the main pulse wave interval larger than 50 ms as shown in Equation (3); “range” is the range of the skin electrical signal and pulse wave signal as shown in Equation (4); “1dmean” is the mean value of the first order difference of the skin electrical signal (Equation (4)); *n*_max_ and *n*_min_ are *n* corresponding to maximum and minimum signal *x*_max_ and *x*_min_, respectively; and *x_th_* is the normalized threshold of the physiological signal features:

(5) $$x_{th}=\sum_{i=1}^{n}(\frac{x_{n}-x_{\min}}{x_{\max}-x_{\min}})/n$$

where *n* is the number of signals, and *x_n_* is the *n*th signal.

The range of the skin electrical signal shows a strong positive correlation between the emotional trends of LAPV and HANV, so the range of the skin electrical signal is used to judge LAPV and HANV. Since the skin electrical signal and pulse wave signal are extracted with the device worn by the fingers, considering the simplicity of the interactive device, the pulse wave and skin electrical signals are selected as the physiological signals of the emotional judgment model.

The program continues when the range of the skin electrical signal is larger than the normalized threshold *x_th_*, otherwise, returning. Then, the program continues if *n*_max_ > *n*_min_, otherwise it is determined as LAPV. Finally, it is determined as HANV if the mean value of the first order difference of the skin electrical signal 1dmean is larger than the normalized threshold *x_th_*, otherwise, returning to range.

The pulse wave signal meeting “BpNN50” goes to the next step. On the one hand, it is determined as LANV if the range of the pulse wave signal is larger than the normalized threshold *x_th_*, otherwise, returning. On the other hand, the program continues when the range of the pulse wave signal is less than the normalized threshold *x_th_*, otherwise, returning. Finally, it is determined as HAPV if “1dmean” > *x_th_*, otherwise, returning.

## 4. Design of Emotion Adaptive Interactive Game

The game Super Mario is adapted through judging the corresponding special events triggered by the player’s emotional trend, which enhances the player’s game experience. The game Super Mario was chosen for the following two reasons: (1) The game is simple to play and easy to operate. It is suitable for players at all levels and can reduce the likelihood that the test results are affected by the participants’ proficiency in the game. (2) Various events in the game that affect the subjects’ emotions are independent, clear, easy to divide, and meet the requirements of our experiment for emotional arousal.

### 4.1. About the Game Super Mario

(1) The core mechanism of the game

The core mechanism of the Super Mario game is to use the keyboard to move on the map while acquiring resources and avoiding enemies. When the player presses the left or right direction keys of the keyboard, the character will move in the corresponding direction. When the player presses the space bar on the keyboard, the character will jump up, and this action can be used to avoid an enemy. If the player jumps up and steps on an enemy’s head, he can destroy the enemy. In addition, the player can also pick up special energy items by jumping and hitting designated props to make the character bigger, move faster, jump higher, or attack an enemy with fireballs.

(2) The gameplay

The Super Mario game is easy to operate, easy to learn, moderate in difficulty, and suitable for all types of players. The content of the game is easy to understand, the rules are clear, the rewards and penalties are clear, and each game event is relatively independent.

(3) The objectives of the game and the expected player experience

The goal of the Super Mario game is to let players experience various events in the game to wake up different emotions. It is easy to cut the emotional change data caused by the event because the triggering environment and conditions of each event in this game are relatively independent. It is expected that players will generate corresponding emotional changes due to various events in the game and recover their emotions to the standard value in the gentle stage between events.

### 4.2. Special Events of the Game

**Figure 3:**
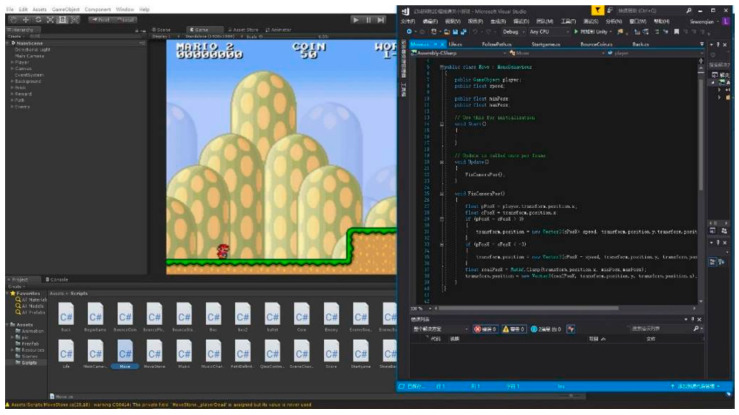
Interactive game based on Unity3D.

**Figure 4:**
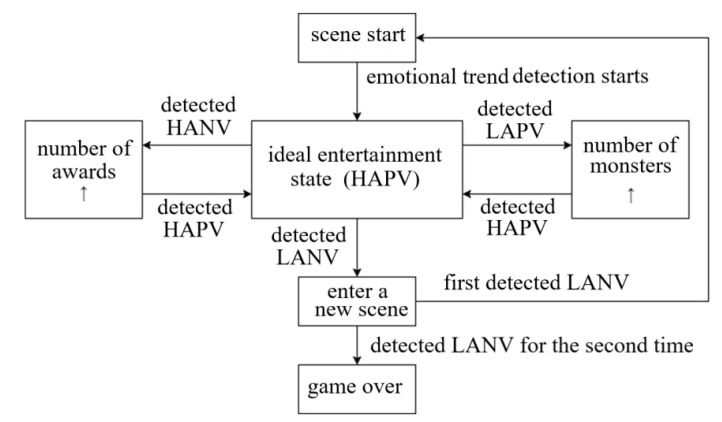
Special event activation conditions.

In the process of game adaptation, all picture materials come from the materials of sharing package in the network [24] as shown in Figure 3. The package was chosen from the network because, first, these pictures can better induce the emotional trend of the subjects, and second, they can enhance the game experience of the subjects. The special events of the game are shown in Figure 4.

When the emotional trend is HANV (high-arousal and negative-valence), the players enter a state of negative-valence. At this time, the game adaptive system will trigger an event that is opposite negative-valence and that can directly affect the emotion, i.e., rewarding the player with a large number of mysterious bricks that can help the character upgrade in order to adjust the player’s emotion from negative-valence to positive-valence. When the emotional trend is LAPV (low-arousal and positive-valence), the players enter a state of low-arousal. At this time, the game adaptive system will trigger an event that is opposite low-arousal and that can directly affect the emotion, i.e., making appear a large number of small monsters that can increase the difficulty of the game and raise the interest of the player in order to adjust the player’s emotion from low-arousal to high-arousal. When the emotional trend is HAPV, the subjects are in an ideal entertainment state without any reaction. When the emotional trend is LANV, the subjects enter a new game scene in order to stimulate the player’s interest and emotion.

The character action is performed using the Vector2.x and Vector2.y functions of Unity2D. The regeneration and activation of the game are controlled using the instantiate function, and whether the subject’s emotion remains in the type of emotional trend is judged using the while loop function.

## 5. Test Results and Analysis

It is easy to directly observe the variation of emotion trend from the waveform of the skin electrical signals, which are recorded with the speed function “Time.deltatime*” and the “Debug.Log()” function for saving the calculation time and accelerating calculation progress. In the process of the calculation, the time length of the calculation segment is kept consistent with the test because the skin electrical signals are varying continuously, and the calculation is carried out every 10 s.

Ten subjects aged between 24 and 30 years were selected for the test. Among the ten subjects, there were six men and four women, and half of the subjects had previously participated in similar tests.

### 5.1. Design of the Emotion Judgment System

**Figure 5:**
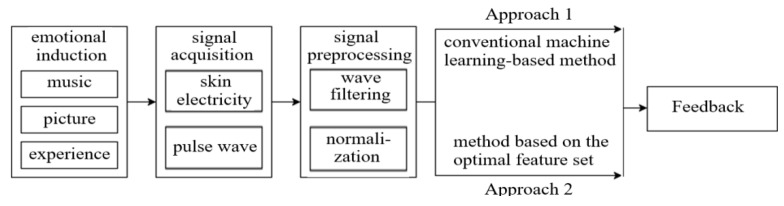
Two approaches of emotion judgment.

In order to illustrate the performance of the emotion judgment system described in Section 3 and compare the effectiveness of the emotion judgment through the physiological signal data, two approaches of emotion judgment were designed in the test as shown in Figure 5.

In Approach 1, the conventional machine learning-based method is used to judge the emotional trend through the physiological signal data, and the data split is set up with 70% as the training set and 30% as the validation set, i.e., introducing:

using Accord.MachineLearning.VectorMachines.Learning;
using Accord.Statistics.Kernels;
and then using the support vector machine toolkit of C# language.

In Approach 2, the features of the signal are separated into a time domain, frequency domain, and feature related with the physiological process. The time domain is determined with 18 pulse wave signals and 24 skin electrical signals. In the frequency domain, the feature mold and computation approach for the signals of skin electricity and pulse wave are similar. The feature related with the physiological process consists of seven skin electrical signals and 10 pulse wave signals. Based on the separation of features, the dimensionality reduction in the original signals is conducted to render the emotional recognition more effective and accurate. Using the PCA, the principal components are obtained, and the weight threshold of each feature of the signal on the principal component is taken as the criterion for the selection of the feature. In the end, the original features, which play the leading role, are defined as a subset of the optimal feature. Based on the subset, the PCC is employed to determine the relation of the optimal features and emotional interval. The PCC is used to compute the features of four emotion trends and extract the significance P of the features. According to the correlation coefficient and significance P, the threshold of the optimal features related to the emotional trends is defined, and the threshold is used to judge the emotional trends. Therefore, the real-time ability and interactivity of Approach 1 and 2 are compared with the tests.

**Figure 6:**
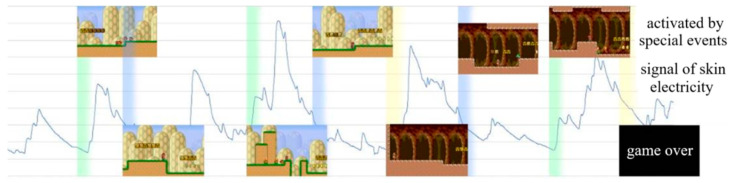
Records of the subjects’ emotions activated by special events.

In the test, the number of the subjects’ emotions that was activated by special events was recorded as shown in Figure 6. Each subject underwent two rounds of tests; Approach 1 and Approach 2 as shown in Figure 5 were used to judge the emotion trend in the first and second rounds of the test, and the results are shown in Table 1 and Table 2, respectively, where the significance test has been performed in the comparisons. In Table 1 and Table 2, the more times the subject is activated by the emotional trend, the sharper the subject’s perception to the change in the emotional trend. For example, in Figure 6, subject 3 is activated eight times by special events as shown in Table 2: two of them enter a new scene and end the game due to the state of negative-valence and low-arousal (LANV), and the other two special events are activated three times each.

### 5.2. Result Analysis

The second round of the test results of the third subject showed that the subject had activated eight special events in total, two of which were due to the low level of pleasure and arousal to enter a new scene and end the game, and the other two special events had been activated three times respectively, i.e., the probability of activation of special events is higher compared with the first round of the test. Other subjects have similar test results, indicating that the emotion judgment system based on the set of optimal signal features is better than that based on the conventional machine learning-based method in interactivity.

Emotion recognition is delayed in most cases, especially at the beginning of the test. There may be two reasons for this. One is that the subject has just started the test and has not yet fully entered the test state. The other is that it takes a certain amount of time to achieve high arousal. However, the recognition results of several emotional trends of most subjects are basically correct, meeting the expectations of the test.

It can be observed from Table 1 and Table 2 that (1) each subject is activated to varying degrees by emotional trends, showing that the emotion judgment system and design of the emotional adaptive interactive game presented in this paper are effective; (2) the number of times that each subject is activated by the emotional trend is different, indicating that the subjects’ perception to the change in the emotional trend is different; (3) the number of times activated by the emotional trend for subjects who participated in the test before are basically larger than that for subjects who did not participate in the test, indicating that the test experience of the subject has an impact on the test results; and (4) the number of times activated by the emotional trend obtained with Approach 2 are larger than that obtained with Approach 1, showing that the emotion judgment system based on the set of optimal signal features is better than that based on the conventional machine learning-based method.

## 6. Conclusions

In order to further study the effectiveness of an emotion judgment system and the effect of a game experience with non-directly controllable signals, a system of emotion recognition and judgment is established, and an emotion adaptive interactive game is designed by adapting the game Super Mario. A total of 10 subjects were selected for the test on the interactive game and emotion judgment system; meanwhile, the results using the emotion judgment system based on a set of optimal signal features and conventional machine learning- based method are compared. The main conclusions are summarized as follows.

(1) The emotion judgment system and design of the emotional adaptive interactive game are effective. The game, which is adapted through judging the corresponding special events triggered by the player’s emotional trend, can enhance the player’s game experience.

(2) In the process of playing the game, the player’s perception to the change in the emotional trend is different, and the test experience of the players has an impact on the test results.

(3) The emotion judgment system based on the set of optimal signal features is better than that based on the conventional machine learning-based method.

## Figures and Tables

**Table 1:** The number of the subject’s activated emotions with Approach 1.

Subjects	Participated in Test before	HANV	LAPV	HAPV	LANV
1	yes	2	1		1
2	yes	3	0		1
3	yes	2	1		2
4	yes	4	1		1
5	yes	0	2		2
total numbers of 1~5		11	5		7
6	No	0	0		0
7	No	3	1		0
8	No	0	1		2
9	No	2	3		1
10	No	2	1		1
total numbers of 6~10		7	6		4
total numbers of 1~10		18	11		11

**Table 2:** The number of the subject’s activated emotions with Approach 2.

Subjects	Participated in Test before	HANV	LAPV	HAPV	LANV
1	yes	2	3		2
2	yes	4	1		1
3	yes	3	3		2
4	yes	3	1		2
5	yes	4	3		0
total numbers of 1~5		16	11		7
6	No	0	0		1
7	No	2	2		0
8	No	3	1		1
9	No	4	2		2
10	No	2	2		2
total numbers of 6~10		11	7		6
total numbers of 1~10		27	18		13

## Data Availability

Data sharing not applicable.
